# Electroporation-Mediated Genome Editing of Livestock Zygotes

**DOI:** 10.3389/fgene.2021.648482

**Published:** 2021-04-13

**Authors:** Jason C. Lin, Alison L. Van Eenennaam

**Affiliations:** Department of Animal Science, University of California, Davis, Davis, CA, United States

**Keywords:** gene editing, zygote, embryo, CRISPR, mosaicism, electroporation

## Abstract

The introduction of genome editing reagents into mammalian zygotes has traditionally been accomplished by cytoplasmic or pronuclear microinjection. This time-consuming procedure requires expensive equipment and a high level of skill. Electroporation of zygotes offers a simplified and more streamlined approach to transfect mammalian zygotes. There are a number of studies examining the parameters used in electroporation of mouse and rat zygotes. Here, we review the electroporation conditions, timing, and success rates that have been reported for mice and rats, in addition to the few reports about livestock zygotes, specifically pigs and cattle. The introduction of editing reagents at, or soon after, fertilization can help reduce the rate of mosaicism, the presence of two of more genotypes in the cells of an individual; as can the introduction of nuclease proteins rather than mRNA encoding nucleases. Mosaicism is particularly problematic in large livestock species with long generation intervals as it can take years to obtain non-mosaic, homozygous offspring through breeding. Gene knockouts accomplished *via* the non-homologous end joining pathway have been more widely reported and successfully accomplished using electroporation than have gene knock-ins. Delivering large DNA plasmids into the zygote is hindered by the zona pellucida (ZP), and the majority of gene knock-ins accomplished by electroporation have been using short single stranded DNA (ssDNA) repair templates, typically less than 1 kb. The most promising approach to deliver larger donor repair templates of up to 4.9 kb along with genome editing reagents into zygotes, without using cytoplasmic injection, is to use recombinant adeno-associated viruses (rAAVs) in combination with electroporation. However, similar to other methods used to deliver clustered regularly interspaced palindromic repeat (CRISPR) genome-editing reagents, this approach is also associated with high levels of mosaicism. Recent developments complementing germline ablated individuals with edited germline-competent cells offer an approach to avoid mosaicism in the germline of genome edited founder lines. Even with electroporation-mediated delivery of genome editing reagents to mammalian zygotes, there remain additional chokepoints in the genome editing pipeline that currently hinder the scalable production of non-mosaic genome edited livestock.

## Introduction

Genome editing offers an opportunity to introduce targeted genetic alterations into livestock genomes. To be useful in animal breeding, these alterations have to be transmissible through the germline. To date, in livestock, this has mostly been achieved by editing somatic cells and subsequently cloning the edited cell line to make an animal (Tan et al., 2016). Somatic cell nuclear transfer (SCNT) cloning remains an inefficient process and limits the genetic diversity of the germplasm to specific cell lines. Editing in zygotes offers an opportunity to introduce alterations to the next generation of a breeding program, and has the advantage of producing a diversity of foundation animals as each zygote will produce a genetically distinct animal, as opposed to animals derived from a clonal cell line (Bishop and Van Eenennaam, 2020). To date, the standard method of delivering genome-editing components into livestock zygotes has been cytoplasmic microinjection (MI). This method requires expensive equipment and is both labor and time intensive, as a highly skilled individual is required to inject zygotes with genome-editing components one-by-one. It can take hours to microinject a large number of zygotes, and this can result in considerable variation in the timing of MI relative to fertilization. Additionally, varying skill levels introduces operator-dependent variation into editing experiments.

**Figure 1:**
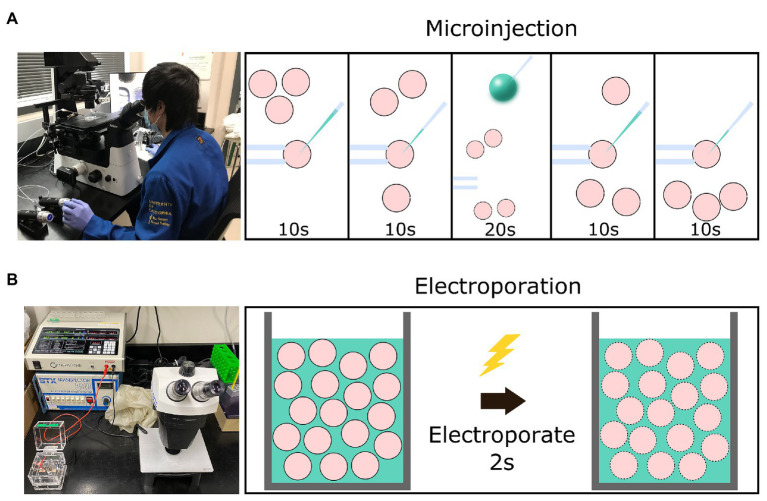
Graphical schematic of a comparison between setup and time necessary for the microinjection vs. electroporation of embryos. **(A)** The equipment necessary for the microinjection of embryos and the workflow involved to introduce editing reagents (green) into four presumptive zygotes (pink) using a holding needle (left) to stabilize the zygote before introducing the injection needle (right). **(B)** The equipment necessary for the electroporation of embryos and the workflow involved to introduce editing reagents into 30–100 presumptive zygotes *via* a cuvette.

Electroporation offers an alternative method of delivering genome-editing components into zygotes. Although electroporation has traditionally been used to introduce reagents into cultured cell lines, it is also effective at introducing editing reagents into mouse and rat zygotes (Peng et al., 2012; Kaneko et al., 2014; Kaneko and Mashimo, 2015; Hashimoto et al., 2016). The protocol for electroporation requires only a stereomicroscope, an electroporator, and an electroporation cuvette. Zygotes are placed into a cuvette or onto a slide while suspended in a medium containing genome-editing reagents (Takemoto, 2020). The electroporator directs pulses of electrical currents through the zygotes *via* electrodes to create temporary micro-holes in the zona pellucida (ZP) and plasma membrane to allow the movement of genome editing reagents into zygotes (Figure 1). The workflow of delivering genome-editing reagents is considerably accelerated relative to MI, as anywhere from 35 to 100 zygotes can be electroporated simultaneously (Modzelewski et al., 2018).

Due to the potential scalability and ease of use of electroporation, it has the potential to become the platform to enable high throughput genome editing in livestock species. However, species specific optimization of electroporation parameters is necessary to achieve both a high survival-rate and efficient editing of zygotes. Here, we review the literature on electroporation-mediated genome editing, with a focus on conditions that maximized zygote survival and editing efficiency in livestock species.

## Electroporation Conditions

One of the first studies published on the electroporation of mouse zygotes concluded that the voltage, pulse length, and concentration of clustered regularly interspaced palindromic repeat (CRISPR) RNA-guided endonuclease Cas9 (Cas9)/single guide RNA (sgRNA) all play a critical role in the survival of embryos and efficiency of mutations (Hashimoto and Takemoto, 2015). The study noted that higher voltages, longer pulse lengths, and higher Cas9/sgRNA concentrations were all positively associated with increased editing efficiency, but negatively correlated with embryo viability. There is a need to strike a balance between the mutation rate and embryo viability when optimizing electroporation conditions. The most efficient parameters for electroporation are highly dependent both on the species of zygote and type of edit (knockout vs. knock-in), therefore, it is necessary to optimize the parameters for each of these variables in order to maximize the generation of live edited animals.

There are several variables to consider when optimizing electroporation conditions including the voltage to be used, how many times that voltage will be applied (number of pulses), and the length (width) of the pulse. There are also two common types of pulses that are often used in electroporation, square-wave, and exponential decay pulses. Square-wave pulses are pulses of a consistent voltage set for a specific amount of time whereas an exponential decay pulse is a continuous pulse with a decaying voltage. In the electroporation of embryos, only square-wave pulses have been reported and there are two sub-types that are commonly used, a “poring” pulse which is a brief mid-level voltage pulse designed to open holes in cell membranes, and a long low voltage “transfer” pulse that is designed to transport negatively charged nucleic acid molecules into cells and nuclei (Sukharev et al., 1992). Combined pulse electroporation uses alternating poring and transfer pulses and can increase the transfection of eukaryotic cells with plasmid DNA or siRNA (Stroh et al., 2010). However, not all electroporators have both pulse types available, and often only the poring voltage is used and reported in many papers.

### Poring Pulse Voltage

Increasing the poring voltage has been shown to increase the density of membrane pores (Gowrishankar et al., 2006; Krassowska and Filev, 2007; Saulis and Saulė, 2012). Studies focused on the electroporation of rat and mouse zygotes have typically reported success in producing genome edited animals when using poring voltages of 25–50 V/mm and anywhere from 2 to 7 pulses (Supplementary Table S1). Poring voltages of 30, 100, and 300 V/mm were tested to find the optimal conditions and 30 V/mm resulted in the highest development and mutation rate in mice. These electroporation experiments achieved mutation rates of 13–100%, suggesting the possibility of high efficiency editing with the further optimization of parameters (Qin et al., 2015). It was noted that higher voltages typically achieved higher mutation rates, although embryo viability was concomitantly decreased.

Studies with livestock zygotes typically report using lower voltages, with porcine zygotes reporting success with 25–30 V/mm and 2–5 pulses; and bovine studies 15–20 V/mm and 2–3 pulses (Supplementary Table S1). Bovine zygotes appear to be especially sensitive to high voltages; with 20 V/mm (three pulses, 1 ms width) resulting in lower blastocyst rates than 10 V/mm (Namula et al., 2019). Increasing the voltage strength to 45 V/mm (five pulses, 3 ms width) was associated with high rates of bovine zygote lysis suggesting damage to the cell membrane lipid bilayer (Wei et al., 2018). Similar results were also reported by Miao et al. (2019), where pulses of 20, 25, and 30 V/mm had an increasingly negative impact on bovine blastocyst development rates. One study found that 15 V/mm achieved significant membrane permeabilization in bovine zygotes to enable efficient rates of gene knockout using Cas9:sgRNA ribonucleoproteins (RNPs), while maintaining acceptable rates of embryo development (Camargo et al., 2020).

### Pulses

Evidence have suggested that pulse number and duration both play a role in the size and density of pores created. Increasing the number of pulses was shown to increase the density of pores, and increasing pulse duration increased the size of the pores created (Gowrishankar et al., 2006; Krassowska and Filev, 2007; Saulis and Saulė, 2012). To test the effect of increasing the number of pulses, Chinese hamster ovary cultured cells were electroporated with a varying number of square-wave pulses. A positive linear relationship was found between the number of pulses and the amount of DNA that entered the electroporated cells (Escoffre et al., 2011). Mouse and rat studies found 2–7 pulses of 1–5 ms pulse widths to be effective in generating efficient mutation and developmental rates. Conditions for electroporating intact rat embryos using zinc finger nucleases (ZFNs), transcription activator-like effector nucleases (TALENs), and the CRISPR associated (Cas) mRNAs were first optimized for the most efficient editing in a study by Kaneko et al. (2014). Using the voltage strength of 45 V/mm, various pulse lengths were examined, and for ZFN, a pulse length of 1.5 ms was the most efficient parameter for generating edited embryos with a survival rate of 91% and editing rate of 73%. Rat embryos electroporated with both TALEN and Cas9 editing reagents showed high survival rates with a pulse length of 2.5 ms, however, the editing rates for these nucleases were only 18 and 9%, respectively, possibly due to the fact that TALEN and Cas9 mRNA are three times larger than that of ZFN mRNA (Kaneko et al., 2014).

Porcine studies have found 4–5 pulses of 1–2.5 ms pulse widths to be successful, and bovine studies have found 2–6 pulses of 1–3 ms pulse widths to be successful (Supplementary Table S1). Various pulse numbers and durations were tested in the electroporation of porcine zygotes, and similar to rodent zygotes, mutation rates increased in proportion with increased pulse numbers and duration, however, blastocyst development rates fell to near zero when the parameters were increased to seven pulses of 3 ms (Tanihara et al., 2016). Nishio et al. (2018) tested a range of voltages as well as unipolar and bipolar pulses, and the results showed that bipolar pulses and voltages over 30 V/mm resulted in significantly lower rates of blastocyst formation, whereas 25 V/mm and unipolar pulses resulted in acceptable embryo survival and editing. Another study by Hirata et al. (2019a) tested the effect of the number of pulses on the blastocyst formation rate and successfully generated edited blastocysts with much higher efficiencies. Both oocytes and zygotes were electroporated at 30 V/mm in this study, and the authors found that using more than five pulses resulted in a significantly lower blastocyst formation rate. The mutation rate varied between electroporation of matured oocytes and putative zygotes, and additionally by the gene being targeted. The same group later followed up with another publication utilizing five pulses at 25 V/mm to generate edited embryos, however, no blastocysts developed so only two to eight cell embryos were analyzed. The authors found that 80–100% of the analyzed embryos showed the intended mutations (Hirata et al., 2019b).

There are currently only five studies describing the electroporation of bovine zygotes to generate knockout embryos. The first of these five studies targeted the Myostatin (*MSTN*) gene to test the effects of voltage strength and electroporation timing on embryo survival and mutation rates. They found that using 20 V/mm considerably lowered the blastocyst formation rate, however, there was a strong correlation between increasing voltage strength and mutation rates. That study also concluded that electroporating bovine zygotes 10 hours post-insemination (hpi) yielded higher mutation rates than electroporating zygotes 15 hpi regardless of the voltage used (Qin et al., 2015; Namula et al., 2019). Another study utilized *in vivo*-derived blastocysts and examined the quality of hatched blastocysts and blastocysts with their ZP still intact after electroporation. The authors concluded that the intact status of a blastocysts’ ZP played a role in the quality of blastocysts as the diameter of the hatched blastocysts shrank significantly after electroporation indicating a loss of quality, whereas the diameter of ZP intact blastocysts did not change significantly after electroporation (Tanihara et al., 2019a). The result supports previous experiments in mice embryos that found the removal of the ZP to potentially hinder embryonic development (Bronson and McLaren, 1970; Modliński, 1970; Chen et al., 2016; Troder et al., 2018; Miao et al., 2019; Tanihara et al., 2019a). Camargo et al. (2020) reported efficient knockout of bovine *OCT4* following electroporation at 17 hpi using six 15 V/mm poring pulses of 1.5 ms at 50 ms intervals and a 10% decay rate of successive pulses. Transfer pulses were set at 3 V/mm, with five pulses of 50 ms at 50 ms interval with a 40% decay rate and positive/negative polarity. In that study, 92.3% of the electroporated embryos evaluated contained the intended edit, however, it should be noted that only a single embryo reached the blastocyst stage under these conditions.

Together, these findings suggest that increasing the duration and number of pulses increases the mutation rates of electroporation-mediated genome editing, correlating with an increase in pore density and size allowing for greater amounts of genome editing components to enter the cells. However, increasing parameters to increase transfection efficiency, and/or weakening the zona pellucida can negatively affect subsequent embryonic development, further demonstrating the need to strike a balance between editing efficiency and embryo viability when optimizing electroporation parameters.

### Concentration of Editing Reagents

The concentration of editing reagents used is yet another parameter that affects the efficiency of electroporation-induced gene editing. Mouse and rat embryos were electroporated with various Cas9 mRNA/gRNA/single-stranded oligonucleotide (ssODN) donor concentrations to optimize conditions for generating knock-in and knockout animals (Kaneko and Mashimo, 2015). The study found that increasing the Cas9 mRNA/gRNA/ssODN concentrations to 400/600/300 ng/μl in both mice and rats resulted in editing efficiencies of 67 and 88%, respectively. Qin et al. (2015) also tested different concentrations of Cas9 mRNA/gRNA and found that increasing the concentrations from 200/100 to 600/300 ng/μl, respectively, increased editing efficiency from 3 to 57% (Qin et al., 2015).

However, when using ssODN donors to optimize conditions for the delivery of a large donor repair plasmid in rat zygotes, it was found that the electroporation of Cas9 protein/gRNA/ssODN at 950/200/200 ng/μl decreased development and did not improve editing efficiency when compared to 475/150/150 ng/μl (Remy et al., 2017). Increasing Cas9 protein and gRNA concentrations from 20 to 100 ng/μl for MI of porcine zygotes increased not only mutation efficiency, but also the proportion of bi-allelic mutations (Tanihara et al., 2019b). A very recent paper tested seven different concentrations of Cas9 protein (0, 25, 50, 100, 200, 500, and 1,000 ng/μl) in porcine zygotes without changing the gRNA concentration of 100 ng/μl, and found that neither embryonic development nor non-specific off-target cutting was affected by Cas9 concentration, although the frequency of biallelic edits tended to increase with Cas9 protein concentration. Additionally, the gene editing efficiency, defined as the frequency of indel mutations in each edited blastocyst, was significantly lower with 25 ng/μl of Cas9 protein compared with higher Cas9 protein (Le et al., 2020). Collectively, these results suggest that, as with voltage and number of pulses, increasing the total concentration of editing reagents is associated with an increase in editing efficiency. Moreover, there appears to be an optimum concentration beyond which embryo viability is impaired with no concomitant increase in editing efficiency, and that may vary depending upon the species and target gene.

## Size of Zygote and Timing of Genome Activation

**Figure 2:**
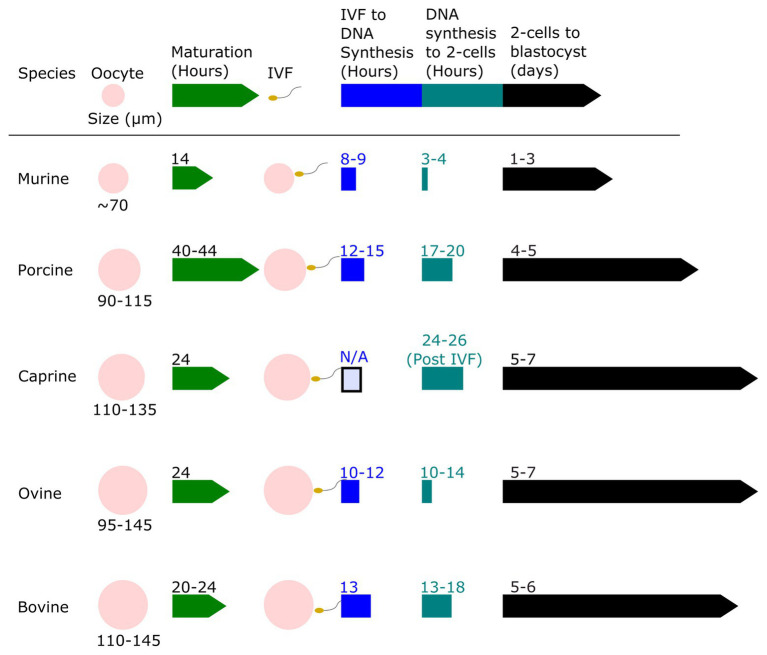
Relative oocyte size and a timeline of embryo development for murine, porcine, caprine, ovine, and bovine zygotes. The oocyte size of murine, porcine, caprine, ovine, and bovine species are shown to scale and compared. The relative timeline of embryo development from the oocyte stage to blastocyst stage after *in vitro* fertilization (IVF) is shown. Data derived from Harlow and Quinn (1982), Motlik et al. (1984), Crosby et al. (1988), Papaioannou and Ebert (1988), Sakkas et al. (1989), Prather (1993), Campbell et al. (1994), Gardner et al. (1994), Laurincik et al. (1994), Fair et al. (1995, 1997), Rath et al. (1995), Serta et al. (1995), Bouniol-Baly et al. (1997), Otoi et al. (1997), Gómez et al. (1998), Wang et al. (1998, 2012), Anderson et al. (1999), Comizzoli et al. (2000), Raghu et al. (2002), Sanfins et al. (2003), Ciemerych and Sicinski (2005), Moon et al. (2005), Griffin et al. (2006), Ptak et al. (2006), Surjit et al. (2006), Zhou and Zhang (2006), Anguita et al. (2007), Chaves et al. (2010), Catalá et al. (2011), O’Hara et al. (2014), Paramio and Izquierdo (2014), Morohaku et al. (2016), Cadenas et al. (2017), Yoon et al. (2018), HosseinNia et al. (2019), McLean et al. (2020), and Owen et al. (2020).

Zygote size is another factor that may influence the efficiency of gene editing using electroporation. Agarwal et al. (2007) found that cell diameter was positively correlated with cell transmembrane potential. This suggests that larger embryos may be permeabilized by a lower voltage than is needed for smaller cells. Figure 2 shows the proportional size of embryos from various mammalian species, ranging from mice (80 μm diameter) to cattle (110–120 μm). In the early embryo, the primary repair mechanism for DNA double-strand breaks (DSBs) is the non-homologous end-joining (NHEJ) repair pathway. The homology directed-repair (HDR) pathway is primarily restricted to actively dividing cells (S/G2-phase), and only becomes highly active toward the end of the first round of DNA replication (Hustedt and Durocher, 2017). It is worth noting that the long G2 phase resulting from genome activation at the two-cell stage in mice is known to be associated with elevated rates of gene knock-ins, presumably due to both the open-chromatin state during genome activation, and the fact that HDR is predominantly active in the late S-G2 phases (Gu et al., 2018; Plaza Reyes and Lanner, 2018). The timing of zygotic genome activation varies among species (Li et al., 2013), ranging from as early as the S/G2 phase in the male pronucleus of the mouse zygote, to the four-cell stage in pigs, the eight-cell stage in goats, and between the eight‐ and 16-cell stages in cattle and sheep (Sirard, 2012; Graf et al., 2014; Deng et al., 2020). It is unclear if the facts that among mammals mice are “early genome activators” while livestock (e.g., bovine) are considered “later genome activators” (Svoboda, 2018), means it is more difficult to achieve gene knock-ins in early livestock embryos.

## Mosaicism and the Timing of Electroporation

Mosaicism is the presence of two or more genotypes in the cells of one individual. Mosaicism poses a problem when generating live animals due to false-positive genotyping, non-transmission of mutations to offspring, and complications with phenotyping (Mehravar et al., 2019). Avoiding mosaicism is particularly important in large livestock species, especially uniparous large animals like cattle with a 2-year generation interval. Whereas researchers utilizing mice can breed mosaic founders and practically guarantee the production of non-mosaic animals with the desired mutations in the first generation (mice reach sexual maturity at 7–8 weeks of age), researchers utilizing livestock may have to wait for years. The ability to generate non-mosaic mutations is therefore essential for the efficient development of genetically modified livestock (Mehravar et al., 2019). Previous studies in mice, cattle, goat, sheep, and pig that have produced genome edited animals using CRISPR and MI have noted the prevalence of mosaic individuals (Hai et al., 2014; Ma et al., 2014; Yen et al., 2014; Oliver et al., 2015; Bevacqua et al., 2016; Zhang et al., 2017). Microinjection with the CRISPR Cas9 system in particular has produced a high rate of mosaic animals (Whitworth et al., 2014; Yen et al., 2014; Sato et al., 2015; Vilarino et al., 2017; Sato et al., 2018; Vilarino et al., 2018).

There are two possible explanations for relative high rates of mosaicism from MI of the CRISPR system. Firstly, the nuclease may continue to target and cut DNA even after the first genomic replication and secondly, genome-editing reagents may have failed to be injected into the zygote until after the first genomic replication. As MI is a long and tedious task, the high rate of mosaicism when producing genome-edited animals using MI may be due to the fact that the zygotes will continue to develop throughout the injection process and while Cas9 is active. The continuous development of zygotes during the MI process results in the later-injected zygotes developing more toward the synthesis stage of the first genomic replication when injected, thus resulting in Cas9 being active later in the zygote stage and past the one-cell stage while the target site remains unmutated (Burkard et al., 2017). Using a gRNA/Cas9 RNP rather than Cas9 mRNA decreases mosaicism as the RNP is active immediately, and does not require the time for mRNA translation and formation of active RNP (Hennig et al., 2020).

A study published in 2016 compared the editing efficiencies of electroporation and MI, and found that electroporation had an 11% lower incidence of mosaicism at an optimized setting when compared to MI, however, the authors electroporated Cas9 protein but injected mRNA, which could have likely played a role in the difference (Chen et al., 2016). Another recent study also evaluated the editing efficiencies in addition to the timing of electroporation and MI of porcine embryos, and found that MI significantly decreased the blastocyst rates in one and two cell injected embryos when compared with electroporation of one cell embryos. The paper used Cas9 protein for both procedures and also noted that mutation efficiency and bi-allelic mutation rate were higher when one cell embryos were microinjected (Le et al., 2021). Additional attempts to further reduce mosaicism have included substituting Cas9 protein for Cas9 mRNA, speeding up the editing process, degrading Cas9 sooner, *in vivo* germline editing, and co-transfection with other reagents such as a three-prime repair exonuclease to improve gene editing efficiency (Chapman et al., 2015; Hashimoto et al., 2016; Tu et al., 2017; Yamashita et al., 2020).

The timing of electroporation also affects the efficiency of generating bi-allelic mutants. Earlier delivery of gene editing components relative to insemination, whether through electroporation or MI, results in an increased rate of bi-allelic and non-mosaic mutants (Vilarino et al., 2017; Namula et al., 2019). One study reported that electroporation of mouse zygotes at only 5 hpi generated 100% non-mosaic animals whereas the electroporation of naturally bred zygotes produced mostly mosaic pups (Hashimoto et al., 2016). The authors concluded that electroporation of mouse zygotes 5 hpi allowed the editing of the mouse genome to occur prior to the first genome-replication and eliminated mosaicism.

In the case of porcine, ovine, and bovine zygotes, DNA synthesis occurs 12–15 hpi, 10–12 hpi, and 18 hpi, respectively (Figure 1). Namula utilized electroporation to deliver CRISPR Cas9 genome-editing components to bovine zygotes and found that electroporation 10 hpi increased the bi-allelic mutation rate, as compared to electroporation at 15 hpi (Namula et al., 2019). Another study in bovine zygotes found a significant reduction in mosaicism rates from MI of zygotes at 10 hpi compared to 20 hpi, however, even the earlier delivery of CRISPR Cas9 genome-editing reagents into bovine MII oocytes did not eliminate mosaicism (Lamas-Toranzo et al., 2019). Microinjection of MII sheep oocytes before fertilization did not eliminate mosaicism, but did produce more bi-allelic mutations compared to MI of zygotes (Vilarino et al., 2017). In pigs, mosaicism was reduced when editing reagents were introduced prior to the onset of DNA replication (Tao et al., 2016). However, the downside of this early electroporation time is that fertilization rates tend to be decreased if oocytes are co-incubated with cumulus cells and spermatozoa for a shorter period of time (Ward et al., 2002).

## Electroporation-Mediated Knockouts

The primary method for DSB repair in gametes and the early zygote is the NHEJ pathway (Rothkamm et al., 2003). Multiple studies in numerous species have used electroporation to deliver CRISPR Cas9 genome-editing reagents into zygotes to generate knockout embryos and animals. Non-mosaic knockouts have been most efficiently produced in rats and mice (Hashimoto et al., 2016; Chen et al., 2019) targeting a wide range of genes, including *LIF* (Kim et al., 2020), *Rad51* (Iwata et al., 2019), and *Rosa26* (Troder et al., 2018).

As previously noted in the poring voltage section, Kaneko et al. (2014) was one of the first to optimize electroporation conditions for rat embryos and successfully generated knockout embryos with a 9% mutation rate. Qin et al. (2015) was able to target 10 different genes in mice and generate 10 different knockout mice with mutation rates from 13 to 100% (Kaneko et al., 2014). Another study published in 2019 utilized Cas12a instead of Cas9 as the nuclease, and targeted three different genes with electroporation. The authors found knockout mutation rates in mouse embryos ranged from 34 to 70% (Dumeau et al., 2019). Unfortunately, mosaicism rates were not studied. More recently, Kaneko explored the possibility of electroporating frozen-warmed pronuclear-stage embryos to generate *Tyr* knockout mice (Nakagawa et al., 2018) and rats (Kaneko and Nakagawa, 2020) using Cas9 protein and dual sgRNA introduced by electroporation after slow freezing. This same group used a combination with electroporation of Cas9 protein and gRNA into rat oocytes following intracytoplasmic sperm injection (ICSI) of frozen or freeze-dried sperm to produce 56 and 50% genome edited offspring for frozen and freeze-dried sperm, respectively (Nakagawa and Kaneko, 2019).

There are currently only a handful of studies describing the generation of live genome edited livestock following electroporation of editing reagents. To date, only porcine and bovine zygotes have been successfully electroporated to produce knockout live animals. Pig researchers have electroporated zygotes and oocytes to generate genome edited blastocysts and live piglets using Cas9 genome editing reagents. A group led by Tanihara has published six studies describing the electroporation of porcine zygotes and efficient editing of blastocysts with at least an 80% success rate in all six studies. They also produced live knockout piglets in three of the studies. The first of the six studies targeted the MSTN gene using five 1 ms pulses at a voltage of 30 V/mm and generated 10 piglets. Nine of the 10 piglets expressed mutations at the target site, seven of which were mosaic. The next study targeted the *TP53* gene using the same parameters which resulted in nine piglets, six of which were genetic knockouts. However, four out of the six mutated piglets were mosaic individuals, a less than ideal outcome if electroporation is to be widely used for the generation of genetically modified livestock (Tanihara et al., 2018). A third study utilized the same parameters again to produce *PDX1* knockout blastocysts, and achieved a success rate of up to 94.1%. That same study also attempted to generate *PDX1* knockout fetuses, however, only one fetus was collected, and it did not carry genetic mutations at the target site (Tanihara et al., 2019c). A subsequent study re-attempted to generate *PDX1* knockout piglets and was successful in producing 10 piglets, nine of which contained the intended knockout. Two of nine piglets with the intended mutations contained no wild-type sequences and another two were mosaic (Tanihara et al., 2020b).

The next porcine study targeted the *CD163* gene with slightly different parameters, using 25 V/mm instead of 30 V/mm, and was able to successfully produce edited blastocysts with a 90% success rate as well as eight piglets, one of which showed a mutation at the intended target (Tanihara et al., 2019d). These studies were able to successfully generate edited blastocysts and piglets, however, up to 40% of the *CD163* blastocysts, four *TP53* piglets, and seven *MSTN* piglets were mosaic. In 2020, this group successfully knocked out (Le et al., 2020; Tanihara et al., 2020a) *MSTN* and *GGTA-1* using electroporation at 12 hpi with five 1 ms transfer pulses at 25V/mm. Five out of six piglets born in the *GGTA1* study carried a bi-allelic mutation in the targeted region of *GGTA1*, with no off-target events (Tanihara et al., 2020a).

Another study published in 2020 attempted to address the issue of generating mostly mosaic mutants through the co-transfection of a three-prime repair exonuclease (Trex2), an exonuclease known to digest DNA ends with breaks. The authors claim to have increased the production of non-mosaic blastocysts by 70.7% when Trex2 was co-transfected with Cas9. Unfortunately, Trex2 is a known inhibitor of HDR which may result in problems if attempting to generate non-mosaic knock-in animals (Yamashita et al., 2020).

Two studies used electroporation to introduce multiple gRNAs to target more than one gene in porcine zygotes. Double bi-allelic mutations were obtained when targeting two genes, although at a low frequency (0–25%) depending upon the gRNA combination (Hirata et al., 2020b). Another study by this group targeted four genes simultaneously. Guides for each gene were first tested independently, and the best guide for each gene was combined to target the four loci. Mutations were observed in one (55.8%) and two genes (20.9%), and no blastocysts had mutations in three or more target genes. This was despite the fact that each guide had independently achieved a rate of at least ~ 20% bi-allelic mutations in blastocysts. The majority of the blastocysts were mosaic. Bi-allelic knockouts were identified in six of the 43 (14%) blastocysts in one of the four genes, and none of these contained edits in a second gene. It is possible that larger than expected deletions or translocations may have occurred that were not detected by the screening methods being used in this study. The authors concluded that the technique to deliver gRNA and Cas9 protein to edit multiple genes will require considerable optimization to improve the success rates (Hirata et al., 2020a).

Miao et al. (2019) published a study describing electroporation of Cas9 protein with gRNA targeting the *Nanos2* gene in mice, pigs, and cattle. They were successful in generating knockout embryos for all three species, and pups in mice. They found that the optimal voltage strengths for efficient survival and editing rates were 20 V/mm for bovine and 30 V/mm for mice and porcine. Analysis of mouse embryos and pups found that two cell embryos were 90% mutated and 70% of pups had a *Nanos2* mutation. Analysis of bovine and porcine embryos revealed bi-allelic *Nanos2* edits at a rate of 82 and 73%, respectively. Some of these knockout *Nanos2* bovine embryos were brought to term, and two calves were born alive, and one was stillborn (Ciccarelli et al., 2020). The stillborn and one live calf were bi-allelic knockouts, while the other live bull calf was mosaic containing both wildtype and mutated allele sequences in varying proportions depending upon the tissue analyzed. It should be noted that electroporation in this study was done at 18–20 hpi.

## Electroporation-Mediated Knock-Ins

While the electroporation of embryos has been able to efficiently generate knockout animals in several species, the generation of knock-in livestock *via* zygote electroporation has not been as widely reported. This can be attributed in part to the low rates of HDR in zygotes, as HDR is predominantly active in the late S-G2 phases of the cell cycle (Liu et al., 2019). This makes it difficult to achieve knock-ins of zygotes.

Knock-in animals require the cleavage of a specific target as well as the integration of donor DNA into the genome. Therefore, in addition to successfully introducing Cas9 and sgRNA and inducing cleavage at the target site, targeted knock-ins also require the successful transfer of template nucleic acid sequences into the zygote. Large supercoiled or linear DNA requires larger functional pores for its entry in the cell compared to short single stranded DNA (ssDNA). Introducing large nucleic acid templates into embryos may require weakening or removing the zona pellucida. The host genome must then be able to repair the cut with the donor template to successfully generate a knock-in embryo. In an unedited cell, the sister chromatid may be used as the homologous donor for HDR; but when generating a knock-in animal, a donor template with the desired insert flanked by homologous arms is necessary to successfully repair the DSB induced by the nuclease and insert the intended sequence (Smirnikhina et al., 2019).

Donor molecules for gene knock-ins include double stranded DNA (dsDNA) as well as ssDNA (Smirnikhina et al., 2019). Double stranded templates have traditionally been used for gene knock-ins; however, ssODN has gained in popularity due to the more rapid construction, higher efficiency, and lower possibility of off-target or plasmid backbone integration (Chen et al., 2011). Additionally, ssODN is able to efficiently integrate into the target locus with homology arms as short as 40 nucleotides, whereas dsDNA donors typically require homology arms around 1–2 kb (Chen et al., 2011; Zhao et al., 2020). Long ssDNA has been used to knock-in large fragments varying from 800 nucleotides to 1.4 kb with efficiencies ranging from 25 to 67% (Quadros et al., 2017). This group used a strategy called efficient additions with ssDNA inserts-CRISPR or Easi-CRISPR (Miura et al., 2018). The homology arms used in that study were 60–105 nucleotides in length. The disadvantage of this approach is that synthesis of long ssDNA greater than 1.5 kb is challenging, and secondary structures could be a problem with long ss templates.

There are also end joining-based techniques that can be used to introduce template sequences into targeted genomic locations. Although NHEJ is the prominent DSB repair pathway, other repair pathways join, anneal, and ligate resected homologous DNA ends. The homology-independent targeted integration method utilizes a donor template containing a gene of interest flanked by the CRISPR Cas9 target sites, but without the use of homology arms. The target sites within the donor template are cleaved alongside the genomic target site, and the gene of interest is inserted by blunt end ligation using the NHEJ repair pathway (Suzuki et al., 2016).

Microhomology-mediated end-joining (MMEJ) is typically defined by homologous joining of sequences less than 25–50 bp in length. A technique called CRISPR/Cas9-based precise integration into the targeted chromosome, or CRIS-PITCh, used an MMEJ donor plasmid containing the knock-in fragment flanked with 40 base pair homology arms and Cas9 RNPs in mouse zygotes to generate knock-ins with efficiencies as high as 40% (Aida et al., 2016).

Targeted integration of linearized dsDNA-CRISPR or tild-CRISPR, uses a linear dsDNA donor template flanked with 800 base pairs of homology arms (Yao et al., 2018). Donor plasmids where the CRISPR target sites are placed outside of 800 bp homology arms so that *in vivo* cleavage by Cas9 generates a linear dsDNA template for homology mediated end joining (HMEJ) have shown robust DNA knock-in efficiency in embryos of several species (Yao et al., 2017). A HMEJ donor plasmid with 800 bp homology arms flanked by the CRISPR Cas9 target site microinjected into bovine zygotes significantly increased the knock-in efficiency of a 1.8 kb fragment when compared to a donor plasmid with the knock-in fragment flanked by 800 bp arms alone (37.0 and 13.8%; *p* < 0.05), and additionally more than a third of the knock-in embryos (36.9%) were non-mosaic. All told, using the HMEJ approach resulted in 7% of total injected embryos being non-mosaic, bi-allelic knock-ins (Owen et al., 2020).

A downside of the HMEJ approach is that the linear dsDNA template, containing the gene of interest and flanking homology arms, generated by Cas9/sgRNA directed cleavage can be inserted into the cleaved genome by blunt end ligation. The lack of control over copy number and orientation of the insert when it is repaired in this way, and the resultant potential presence of random indels and insertion of plasmids into the genome, limits the use of this approach as a precise genome engineering strategy (Salsman and Dellaire, 2017).

## Electroporation of Donor Repair Nucleic Acid Sequences

Grabarek et al. (2002) was the first to demonstrate that nucleic acids can be delivered to isolated oocytes and zygotes by electroporation if the zona pellucida was weakened by exposure to acid Tyrode’s solution. Of relevance to this review is the size of the donor template that can be introduced into zygotes using electroporation. Larger donor plasmids have traditionally been delivered to the zygote *via* MI. There have been only a few studies describing the successful delivery of ssODN donors of 30–200 nucleotides, and even fewer describing the successful delivery of large plasmids into an embryo when using electroporation (Kaneko and Mashimo, 2015; Chen et al., 2016; Hashimoto et al., 2016; Wang et al., 2016; Remy et al., 2017; Bagheri et al., 2018; Troder et al., 2018; Chen et al., 2019).

The majority of knock-in animals created through electroporation have been mice or rat zygotes electroporated with Cas9/gRNA/ssODN. Hashimoto and Takemoto (2015) were able to use an ssODN donor template of 117 nucleotides to disrupt the expression of mCherry in mice. All 11 of the surviving embryos did not fluoresce suggesting a successful knock-in. However, further sequencing did reveal some mosaicism in the edited embryos as up to three distinct alleles were found (Hashimoto and Takemoto, 2015).

Electroporation of an ssODN donor enabled successful genome editing of both mice and rats harboring a single amino acid substitution, with a success rate of 33% in both species (Kaneko and Mashimo, 2015). Other successful electroporation mediated knock-ins include a 92 nucleotide ssODN targeting the *Tyr* gene in mice. In this study, a pulse width of 1 ms produced 47% *Tyr*-edited mice of which 42% were mosaic while a pulse width of 3 ms produced 97% *Tyr*-edited of which 9.4% were mosaic (Chen et al., 2016). Others include a 103 ssODN donor targeting the *Fgf10* gene (Hashimoto et al., 2016), and a 128 bp oligonucleotide targeting the *Aicda* gene (Wang et al., 2016).

Sakurai et al. (2020) utilized oocytes from transgenic mice expressing maternal Cas9 (maCas9) to generate gene-edited embryos and pups. The group compared mutation rates between embryos and pups following zygote transfections either with gRNA alone or with both Cas9 and gRNA. They found that the electroporation of Cas9-expressing transgenic zygotes with gRNA alone was able to generate indels at the target region in nearly 100% of the embryos analyzed, and no off-target mutations were observed. They also found that the electroporation of zygotes expressing maCas9 with gRNA alone showed significantly lower mosaicism rates when compared to wild-type zygotes electroporated with Cas9/gRNA. Most notably, the authors found that the electroporation of maCas9 zygotes with gRNA to disrupt *Et1* resulted in 40% genome-edited pups, compared to wild-type zygotes electroporated either with Cas9 mRNA/gRNA (21%) or Cas9 protein/gRNA (23%).

In this same study, birth rates were also higher following electroporation of maCas9 zygotes. The authors attempted a knock-in mutation at the *Klf5* locus either into maCas9 zygotes with gRNA/ssODN which gave a 48% rate of live pups, as compared to 20–21% for wild-type zygotes electroporated with Cas9/gRNA and ssODN. Similarly, when knock-in mutations were attempted at the *Ar* locus, blastocyst rates for maCas9 zygotes were higher (69%) when compared to wild-type zygotes electroporated with Cas9/gRNA/ssODN (8–15%). Actual knock-in rates at the *Klf1* locus were similar between maCas9 zygotes (46–48%) and wild-type zygotes (41–44%); and knock-in rates at the *Ar* locus were 8% in maCas9 zygotes and 0% in control zygotes.

There is one publication reporting a successful knock-in with bovine zygotes using electroporation, however, it is unknown what the target locus was, or the size of the ssODN template. The publication only details that an ssODN was used as a donor template and that one of 16 blastocysts (6%) collected and analyzed showed a successful knock-in. The authors concluded this result demonstrated that knock-ins are possible with the electroporation of bovine zygotes albeit at a low rate (Wei et al., 2018). The authors also found that a 4.7 kb pEGFP plasmid could only be introduced into bovine zygotes following removal of the zona pellucida using pronase. They reported that only zona-free zygotes generated EGFP-positive blastocysts following electroporation, indicating that the zona pellucida presents a strong barrier for large dsDNA-uptake following electroporation. They concluded that the bovine zona pellucida effectively blocked the delivery of plasmids to the cytoplasm.

In rat and mouse embryos, a 5.1 kb plasmid was successfully delivered into the cytoplasm by electroporation but only following MI of the plasmid, along with all of the CRISPR Cas9 genome-editing reagents, into the sub-ZP space (Bagheri et al., 2018). All mutant blastocysts were found to be mosaic. Although MI of all CRISPR components prior to electroporation allows the donor plasmid to bypass the ZP and integrate into the host genome, this method does not eliminate the high skill and time required to perform MI. A different study attempted to knock-in a 3.1 kb plasmid into the *Rosa26* locus of rats without the use of prior MI, but failed to generate any embryos with successful integration (Remy et al., 2017).

Laser zona drilling (LZD) is another method of facilitating movement across the ZP that may be able to help in the transfection of larger plasmids into zygotes. LZD generates a hole in the membrane of the ZP allowing larger molecules to enter the sub-ZP space and was previously used to assist in the microinjection of CRISPR Cas9 genome-editing components (Bogliotti et al., 2016). Additionally, LZD has been shown to have minimal effects on embryo viability when used in conjunction with MI. LZD in conjunction with electroporation may be able to better facilitate the movement of large plasmids into embryos where the zona pellucida presents a barrier to transfection. However, LZD again requires handling each zygote individually and a high level of skill.

Recombinant adeno-associated viruses offer an opportunity to overcome the size limitation of ssODN donors for knock-in animals. They are relatively small viruses of about 20 nm belonging to the family Parvoviridae that do not incorporate into the host chromosomes. They can however diffuse across the zona pellucida to transiently deliver genes to fertilized mammalian zygotes with intact zona pellucida (Mizuno et al., 2018; Romeo et al., 2020). They have been used to successfully generate genome edited mouse pups without the need for micromanipulation, with both high embryo survival and editing rates (Yoon et al., 2018). A 2019 study used rAAV to transfect large HDR donors of up to 4.9 kb, prior to electroporation with genome editing reagents (Chen et al., 2019). Known as CRISPR RNP electroporation and AAV donor infection (CRISPR READi), the authors generated large DNA fragment knock-in mice by incubating rAAV packaged with ssDNA with zygotes for 6 h prior to electroporation, then cultured and transferred the edited embryos into surrogate mothers (Chen et al., 2019). This technique achieved up to 50% knock-ins, however, the animals had high rates of mosaicism. rAAV-serotypes 1, 2, and 6 have all been used to transduce mammalian embryos of various species, with serotype 6 appearing to be useful in a variety of mammals (Mizuno et al., 2018). Since the AAV genome can be episomally maintained for an extended period, mosaicism might result from insertions that occur after the one-cell stage of embryo development (Mizuno et al., 2018), posing a potential mosaicism issue for livestock applications.

## Discussion

**Figure 3:**
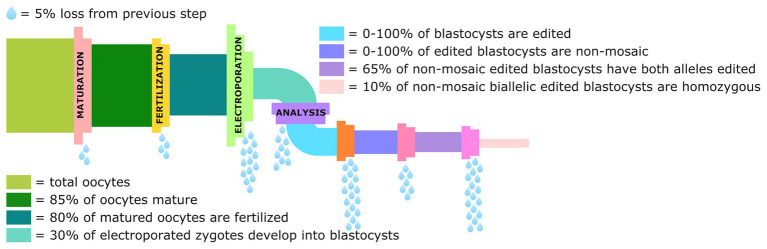
Graphical representation of the losses in the genome editing pipeline from collection of oocytes to the percentage of blastocysts that are non-mosaic homozygotes for the intended edit. Data derived from Remy et al. (2017), Teixeira et al. (2018), and Miao et al. (2019).

The studies done in rodents show the potential that electroporation has to streamline the process of generating genetically modified livestock and making this technology more accessible to laboratories lacking MI expertise. However, the limited number of studies done in cattle and pigs shows much work still remains to optimize these experimental protocols to improve both editing and survival efficiency, and eliminate the production of mosaic animals. There are several chokepoints in the pipeline from the collection of oocytes to the production of non-mosaic blastocysts homozygous for the intended edit, that need to be streamlined and optimized before this technique can become routine (Figure 3).

It is perhaps not obvious to those not working in the field, but a source of livestock oocytes has to be readily available to perform zygote editing, often obtained from ovaries collected at a local slaughter facility. To produce viable mammalian offspring, it is also necessary to have a ready supply of synchronized recipient or surrogate females. This is not an inexpensive undertaking in the case of large livestock species, and due to seasonal breeding and other climatic factors, it is almost impossible to conduct this work during certain times of the year. To improve the efficiency of the process, ideally only blastocysts carrying the desired edits would be transferred to surrogate females. Although studies have shown that taking a biopsy from the trophectoderm of *in vitro* matured bovine embryos can result in live, healthy offspring (de Sousa et al., 2017), a high level of skill is required. Another problem with preimplantation biopsies is that mosaicism decreases the usefulness of these results (Vilarino et al., 2018) as the trophectoderm may have a different genetic composition compared to the inner cell mass.

It is perhaps ironic given the important role that sheep played in the development of livestock genetic engineering and SCNT cloning techniques, that there are currently no published studies detailing electroporation-mediated genome editing of sheep zygotes. All small ruminant edits have been accomplished by either SCNT or embryonic microinjection (Kalds et al., 2020). Future sheep and goat experiments will first need to optimize electroporation conditions prior to generating genetic knockouts and knock-ins, but previous work, especially in cattle, should help pave the way. There are already a number of targets in the sheep and goat genome that have previously been edited using MI of CRISPR Cas9 genome-editing reagents, so the transition to electroporation should be relatively straightforward.

Gene knockouts using the NHEJ pathway have been the most successful type of embryo-mediated genome edit, to date, and there are several experiments documenting very high rates of bi-allelic mutation using electroporation. Although it should be noted that gene compensation through exon skipping has been observed to reinitiate transcription and translation, which can result in partial gain-of-function alleles rather than the predicted nonsense or missense alleles (Lalonde et al., 2017; Smits et al., 2019; Hosur et al., 2020) When the editing reagents are working well and producing 100% bi-allelic knockouts, transferring edited embryos carries little downside. However, if rates decrease below this, the probability of transferring mosaic, hemizygous, or wild type animals increases. Obtaining a high proportion of bi-allelic knockouts of multiple genes in a zygote is still extremely challenging. Likewise obtaining targeted gene knock-ins in zygotes is very inefficient, especially for large DNA insertions. Undoubtedly, further improvements in editing reagents such as base pair editors, and improved repair templates will be forthcoming. Viral transduction using rAAV offers an opportunity to introduce single-stranded DNA of up to 4.5kb in length (Kaulich et al., 2015), although this approach has not yet been applied to livestock zygotes.

Other approaches to increasing the production of non-mosaic edited animals include editing embryonic stem cells (ESCs). The production of porcine (Gao et al., 2019), bovine (Bogliotti et al., 2018), and ovine (Vilarino et al., 2020) stable, pluripotent ESCs have recently been reported. The advantage of using ESCs is that multiple sequential edits could be performed due to their perpetual ability to self-renew. It may be that cloning ESCs increases the efficiency of cloning success relative to SCNT (McLean et al., 2020). Alternatively, embryo complementation or injecting donor totipotent edited stem cells into genome edited knockout, germline ablated host embryos (Ciccarelli et al., 2020; Miura et al., 2020), or edited primordial germ cells in the case of poultry (Woodcock et al., 2019), may provide an alternative approach to produce animals that transmit gametes derived solely from an edited cell line. This could help to resolve the problem of mosaicism that is frequently associated with electroporation-mediated genome editing of mammalian zygotes. The downside of ESCs is similar to SCNT in that they represent a limited genetic pool, and they may accumulate mutations during culture. Delivery of genome editing components into the zygote edits the next generation of a livestock breeding program, and avoids the inefficiencies associated with SCNT. It has been successfully used to achieve targeted knockouts in embryos, although mosaicism can reduce germline transmission, and efficient gene knock-ins have proven difficult. Although electroporation provides an improved approach over MI to rapidly introduce editing reagents into developing zygotes of mammalian food animal species, further development and optimization of enabling methodologies will be required to routinely obtain non-mosaic knockout and targeted-gene insertion founders in livestock at scale. Such developments will be required before genome editing can be seamlessly introduced into livestock genetic improvement programs.

## Author Contributions

JL performed the literature review and authored the first draft of the manuscript. AV edited and provided suggestions. All authors contributed to the article and approved the submitted version.

### Conflict of Interest

The authors declare that the research was conducted in the absence of any commercial or financial relationships that could be construed as a potential conflict of interest.
