# Supplementary material for: Electroporation-Mediated Genome Editing of Livestock Zygotes
Source: Front Genet. 2021 Apr 13;12:648482. doi: 10.3389/fgene.2021.648482 (PMC8078910; doi:10.3389/fgene.2021.648482)
Supplement: Supplementary file 1 [file Table_1.pdf]

SUPPLEMENTARY MATERIAL

Supplemental Table 1. Summary of experiments using electroporation to introduce genome editing reagents into mammalian zygotes. Summary includes: Species, form and type of nuclease used for editing, method of fertilization, electroporation conditions and timing relative to fertilization, type of edit, survival rate, developmental rates and editing efficiency. RNP, ribonucleoprotein; ND, not discussed; IVF, in vitro fertilization; Mate, natural mating; Settings, Electroporation Voltage (V/mm) Pulse length (msec) # pulses; Reagent, medium used for electroporation.

	**Species<br>(Oocyte = *)<br>(Embryo = †)**	**Nuclease**	**RNP, mRNA**	**Electroporation timing<br>Fertilization**	**Settings<br>Reagent**	**Target<br>Type of edit<br>(Size of donor or insert bp or nt)**	**Zygote survival rate**	**Development rate<br>(2-cell = *)<br>(blast = †)**	**Edited rate<br>(live animal = *)<br>(Embryo = †)**	**Reference**
**RODENT**	Mouse†	Cas9	Maternal	IVF	20V<br>3msec<br>5 pulses	Et-1, Tyr<br>KOKO<br>*Klf5, Ar*<br>KIKI<br>*Adm, Amy,*<br>*Aldh2*<br>*Cyp1a1*<br>*Hprt*<br>*Npr3*<br>*Ramp1*<br>*Ramp3*		32-58%*<br>8-69%†	46-48%*	(Sakurai et al., 2020)
	Mouse†<br>Rat†	Cas9	Protein/ dual RNA	ND<br>Mouse: IVF<br>Rat: Mate	Poring:<br>40V<br>3.5msec<br>4 pulses<br>Opti-MEM	*Tyr*<br>KO	Mouse:100%<br>Rat: 100%	Mouse: 100%*<br>Rat: 92%*	Mouse: 18%*<br>Rat: 100%*	(Kaneko and Nakagawa, 2020)
	Mouse†	Cpf1	Protein/ mRNA	ND<br>Mate	ND<br>Opti-MEM	Leukemia Inhibitory Factor<br>*Lif* KO	Protein: 71.6%<br>mRNA: 76.4%	Protein: 84.6%†<br>mRNA: 68.1%†	Protein: 45.5%*<br>mRNA: 33.3%*<br>Protein: 18.1%†<br>mRNA: 13.3%†	(Kim et al., 2020)
	Mouse†<br>In-vivo	Cas9	protein	ND<br>IVF	Poring:<br>50V<br>5msec<br>3 pulses<br>Opti-MEM	*Adamts20*<br>PN-locus<br>*Rad51*<br>Inversion<br>Deletion			Adamts20: 10%*<br>PN-locus: 50%*<br>Rad51: 50%*	(Iwata et al., 2019)
	Rat†	Cas9	Protein/dual RNA	5hr<br>IVF	Poring:<br>20, 30, 40V<br>3.5msec<br>4 pulses<br>Opti-MEM	*Tyr*<br>KO	94-100%	0-25%<br>(Offspring)	0-100%*	(Nakagawa and Kaneko, 2019)
	Mouse†<br>Both in-tact ZP and weak ZP	Cas12a	Protein	ND<br>Mate	Poring:<br>30V<br>3msec<br>6 pulse<br>Own Mix	*UBN1*<br>*UBN2*<br>*RBM 12*<br>KO	72-100%	21-46%†	34-70%†	(Dumeau et al., 2019)

	Species<br>(Oocyte = *)<br>(Embryo = †)	Nuclease	RNP,<br>mRNA	Electroporation timing<br>Fertilization	Settings<br>Reagent	Target<br>Type of edit<br>(Size of donor or insert bp or nt)	Zygote survival rate	Development rate<br>(2-cell = *)<br>(blast = †)	Edited rate<br>(live animal = *)<br>(Embryo = †)	Reference
**RODENT**	Mouse†<br>Weakened ZP	Cas9	Protein	ND<br>Mate	30V<br>3msec<br>6 pulses<br>Opti-MEM	*Tyr*<br>*Sox2*<br>*Rosa 26*<br>KI<br>(Up to 4.9kb)		41%<br>(morula)	18-40%*<br>33-69%†	(Chen et al., 2019)
	Mouse†<br>Both intact and weakened ZP	Cas9	Protein	ND<br>Mate/ IVF	30V<br>3msec<br>2 pulses<br>Opti-MEM	*NPHS2*<br>*ATP1a1*<br>*GTRosa26Sor*<br>*Tmem218*<br>KI/ KO<br>(150nt)			23%*<br>(Unable to knock-in large plasmid)	(Troder et al., 2018)
	Mouse†	Cas9	Protein	ND<br>Mate	Poring:<br>40V<br>3.5msec<br>4 pulse<br>Opti-MEM	*BRCC3*<br>*Vash1*<br>*Vash2*<br>*CTSE*<br>*MCT8*<br>KO/KI		89-97%<br>(Transferred embryos)	60-100%*	(Teixeira et al., 2018)
	Mouse†	Cas9	Protein	6.5-7.5hr<br>IVF	25/30V<br>3msec<br>7 pulses<br>Opti-MEM	*Tyr*<br>*IL11*<br>*SPP1*<br>KI/ KO		90-100%*	50-100%*	(Nakagawa et al., 2018)
	Mouse†<br>ZP pierced, injected	Cas9	Protein	ND<br>Mate	25V<br>3msec<br>7 pulse<br>Own Mix	*Nanog*<br>*RP113a*<br>KI inject<br>9.7kb/7.17kb	90%		2.3-3%†<br><br>KI	(Bagheri et al., 2018)
	Rat†	Cas9	Protein	ND<br>Mate	Various<br>PBS	*EPHX2*<br>*FLNA*<br>*Rosa26*<br>KI<br>(100nt; 119nt)	30-100%		0-100%†	(Remy et al., 2017)
	Mouse†<br>Weakened ZP	Cas9	Protein	ND<br>IVF/ Mate	30V<br>1msec<br>2 pulses<br>ND	*Aicda*<br>*Smc1b*<br>*Rosa 26*<br>KI<br>(34bp insertion)			14-100%*	(Wang et al., 2016)
	Mouse†	Cas9	Protein	5hr<br>IVF/ Mate	30V<br>3msec<br>7 pulses<br>Opti-MEM	*FGF10*<br>KI/ KO<br>(103nt)	93-96%<br><br>2-cell	63.7-100%	44.4%†	(Hashimoto et al., 2016)

	**Species<br>(Oocyte = *)<br>(Embryo = †)**	**Nuclease**	**RNP,<br>mRNA**	**Electroporation timing<br>Fertilization**	**Settings<br>Reagent**	**Target<br>Type of edit<br>(Size of donor or insert bp or nt)**	**Zygote survival rate**	**Development rate<br>(2-cell = *)<br>(blast = †)**	**Edited rate<br>(live animal = *)<br>(Embryo = †)**	**Reference**
**RODENT**	Mouse†<br>Weakened ZP	Cas9	Protein	ND<br>Mate	30V<br>3msec<br>2 pulses<br>Opti-MEM	*Tyr*<br>*Cdh1*<br>*Cdk8*<br>*Kif11*<br>*MecP2*<br>*Sox2*<br>KI/ KO<br>(92nt donor)		18-63%<br>(morula)	27-88%*<br>54-100%†	(Chen et al., 2016)
	Mouse†<br>Weakened ZP	Cas9	mRNA	ND<br>Mate	30V<br>1msec<br>2 pulses<br>Opti-MEM	*Tet1, Tet2*<br>*Cd69,*<br>*Cd226Cd226*<br>*Clec16a*<br>*Cyp27b1*<br>*Fut2*<br>*Ormdl3*<br>*Rgs1*<br>*Tlr7*<br>*Tlr8*<br>*Tnfsf9*<br>KO<br>KI<br>(126nt donor)			0-100%*	(Qin et al., 2015)
	Mouse/ rat<br>Pronuclear Embryo<br>No ZP	Cas9	mRNA	ND<br>Mouse: IVF<br>Rat: Mate	Poring:<br>45V<br>2.5msec<br>4 pulses<br>PBS	*Il2rg*<br>KI/ KO		73-98%*	33-88%*	(Kaneko and Mashimo, 2015)
	Mouse†	Cas9	mRNA	ND<br>Mate	30V<br>3msec<br>7 pulses<br>Opti-MEM	*FGF10*<br>*Rosa26*<br>KI/ KO<br>(117nt donor)	94-95%	51-72%<br>(Embryo)	12-97%†	(Hashimoto and Takemoto, 2015)
	Rat*<br>Embryo	ZFN<br>TALEN<br>CRISPR	mRNA	ND	Various<br>PBS	*Il2rg*<br>KO	24-97%	6-55%<br>(offspring)	4-75%*	(Kaneko et al., 2014)
	Mouse various embryonic stages<br>Weakened ZP			ND<br>Mate	Various<br>Opti-MEM	*OCT4*	85-94%			(Peng et al., 2012)

	Species<br>(Oocyte = *)<br>(Embryo = †)	Nuclease	RNP,<br>mRNA	Electroporation timing<br>Fertilization	Settings<br>Reagent	Target<br>Type of edit<br>(Size of donor or insert bp or nt)	Zygote survival rate	Development rate<br>(2-cell = *)<br>(blast = †)	Edited rate<br>(live animal = *)<br>(Embryo = †)	Reference
**PORCINE**	Pig†	Cas9	Protein	20hr<br>IVF	Poring:<br>45V<br>2.5msec<br>4 pulses<br>Opti-MEM	*GHR*<br>KO		C9: 38%†<br>T: 45%†	C9: 37%†<br>T: 45%†	(Yamashita et al., 2020)
	Pig†	Cas9	Protein	12 & 24hr<br>IVF	Poring:<br>25V<br>1msec<br>5 pulses<br>Nuclease-Free Duplex Buffer	*B4GALNT2*<br>KO		8.1-32.6%†	20-90%†	(Le et al., 2021)
	Pig†	Cas9	Protein	12hr<br>IVF	25V<br>1msec<br>5 pulses<br>Opti-MEM	*CD163*<br>KO			12.5%*<br>84.6-90%†	(Tanihara et al., 2019d)
	Pig†	Cas9	protein	18-20hr<br>IVF	30V<br>3 pulse<br>3msec<br>Opti-MEM	*Nanos2*<br>KO		9-36%†	63-90%†	(Miao et al., 2019)
	Pig†*	Cas9	protein	13hr<br>IVF	30V<br>1msec<br>Various pulses<br>Opti-MEM	*MSTN*<br>*FGF10*<br>KO	52.6-90.7%	8.8-27.6%†	12.5-60%†	(Hirata et al., 2019a)
	Pig†	Cas9	Protein	13hr<br>IVF	20V<br>1msec<br>5 pulses<br>Opti-MEM	PERV pol<br>KO	74-88.9%	0-22%†	80-100%†	(Hirata et al., 2019b)
	Pig†	Cas9	Protein	7hr<br>IVF	25 V<br>1msec<br>5 pulses<br>Opti-MEM	*IL2RG/GHR*<br>KO		20-30%†	85-95%†	(Hirata et al., 2020b)
	Pig†	Cas9	Protein	13hr<br>IVF	25 V<br>1msec<br>5 pulses<br>Opti-MEM	*CMAH/GHR/*<br>*GGTA1/PDX1*<br>KO		29.7%	76.7%†<br>20.9%† (in two target genes)	(Hirata et al., 2020a)
	Pig†	Cas9	Protein	12hr<br>IVF	30V<br>1msec<br>5 pulses<br>Opti-MEM	*PDX1*<br>KO		10.3-11.9%†	0%*<br>37.8-94.1%†	(Tanihara et al., 2019c)

	Species (Oocyte = *) (Embryo = †)	Nuclease	RNP, mRNA	Electroporation timing Fertilization	Settings Reagent	Target Type of edit (Size of donor or insert bp or nt)	Zygote survival rate	Development rate (2-cell = *) (blast = †)	Edited rate (live animal = *) (Embryo = †)	Reference
**PORCINE**	Pig†	Cas9	Protein	12hr<br>IVF	30V<br>1msec<br>5 pulses<br>Opti-MEM	*TP53*<br>KO			66.67%*<br>72.7-100%†	(Tanihara et al., 2018)
	Pig†	Cas9	mRNA	13hr<br>IVF	Various<br>Opti-MEM	*FGF10*<br>KO	61.1-91.4% cleaved	4.1-26.4%†	3.6-7.7%†	(Nishio et al., 2018)
	Pig†	Cas9	mRNA<br>Protein	13hr<br>IVF	30V<br>1msec<br>5 pulses	*FGF10*<br>*MSTN*<br>KO		10-16%†	90%*<br>23-100%†	(Tanihara et al., 2016)
	Pig†	Cas9	Protein	12hr<br>IVF	25V<br>1msec<br>5 pulses<br>Nuclease-Free Duplex Buffer	*GGTA1*<br>KO		Approx. 18%†	83.3%*<br>37.5%†	(Tanihara et al., 2020a)
	Pig†	Cas9	Protein	5hr?<br>IVF	30V<br>1msec<br>5 pulses<br>Nuclease-Free Duplex Buffer	*PDX1*<br>KO		Approx. 15%	90%*<br>76.5-77.8%†	(Tanihara et al., 2020b)
	Pig†	Cas9	Protein	12hr<br>IVF	25V<br>1msec<br>5 pulses<br>Nuclease-Free Duplex Buffer	*MSTN*<br>KO		65.6-78.9%*<br>13.3-23.1%†	Approx. 70-90%	(Le et al., 2020)

	Species (Oocyte = *) (Embryo = †)	Nuclease	RNP, mRNA	Electroporation timing Fertilization	Settings Reagent	Target Type of edit (Size of donor or insert bp or nt)	Zygote survival rate	Development rate (2-cell = *) (blast = †)	Edited rate (live animal = *) (Embryo = †)	Reference
**BOVINE**	Cattle<br>In-vivo derived blastocysts			8 d in vivo–derived blastocysts	20V<br>1msec<br>3-10 pulses<br>Opti-MEM					(Tanihara et al., 2019a)
	Cattle†	Cas9	protein	10/15hr<br>IVF	10, 15, and 20V<br>3 pulse<br>1 msec<br>nuclease- free duplex buffer	*MSTN*<br>KO	60-92%	14-38%†	4.8-16.7%†	(Namula et al., 2019)
	Cattle†	Cas9	protein	18-20hr<br>IVF	20V<br>2 pulse<br>3msec	*Nanos2*<br>KO		9-36%†	63-90%†	(Miao et al., 2019)
	Cattle	Cas9	Protein	18-20hr<br>IVF	20V<br>2 pulse<br>3msec<br>Opti-MEM	*Nanos2*<br>KO			16 embryos,<br>8 pregnancies,<br>3* born (1 stillborn);<br>2 KO; 1 mosaic	(Ciccarelli et al., 2020)
	Cattle†<br>Both intact and weakened ZP	Cas9	protein	8hr<br>IVF	30V<br>6 pulses<br>Opti-MEM	ND<br>Undisclosed KI		33% †	6%†	(Wei et al., 2018)
	Cattle†	Cas9	Protein	17-18 hr<br>IVF	15V (0-30)<br>1.5msec<br>6 pulses<br>Opti-MEM	*ZFX*<br>*OCT4*<br>KO		Approx. 80%*	92.3%†	(Camargo et al., 2020)
